# Knowledge of Dental Students and Practitioners About Medication-Related Osteonecrosis of the Jaw in the Central Region of Saudi Arabia

**DOI:** 10.7759/cureus.52165

**Published:** 2024-01-12

**Authors:** Meshal M Alghofaili, Syed Fareed Mohsin, Nada Mohammed Nahari, Tamim S Alkhalifah, Rayan Khaled Almazyad, Mohammed K Alsaegh

**Affiliations:** 1 Dentistry, Qassim University, Ar Rass, SAU; 2 Oral Maxillofacial Surgery and Diagnostic Sciences, Qassim University, Ar Rass, SAU; 3 General Dentistry, King Khalid University, Abha, SAU

**Keywords:** dental practitioners, antiresorptive/antiangiogenic, dental students, medication-related osteonecrosis of the jaw, bisphosphonates

## Abstract

Background: Bisphosphonates (BPs) are often used in treating benign and malignant disorders. Medication-related osteonecrosis of the jaw (MRONJ) is a significant problem that arises from the long-term use of BPs.

Objective: In this study, we assessed the knowledge of students and dentists about MRONJ in the central region of Saudi Arabia.

Methods: A cross-sectional study was conducted to collect information from dental students and practitioners from the central region of Saudi Arabia. A valid, reliable, and structured questionnaire was used to gather data using a non-probability convenient sampling technique. IBM SPSS Statistics for Windows, Version 22.0 (Released 2013; IBM Corp., Armonk, New York, United States) was used to analyse the data. The descriptive data were expressed as frequencies and percentages to evaluate the association between dentists and students concerning overall knowledge related to osteonecrosis of the jaw, and a chi-squared test was applied.

Results: In total, 250 individuals completed the questionnaire. The general knowledge of antiresorptive/antiangiogenic medications showed that most dentists (87.5%) and students (68.4%) knew about BP medications. A general lack of understanding about the therapeutic uses of antiangiogenic and antiresorptive medications was demonstrated by the participants. A significant proportion of dentists (58.8%) and students (50.9%) were not convinced that invasive dental procedures can be safely performed on patients receiving intravenous BP therapy. A significant proportion of the participants in the sample were unclear of the principal diseases that antiresorptive and antiangiogenic medications target. A mere 22% of respondents were aware of the accurate definition of medications-related MRONJ.

Conclusion: There is insufficient knowledge about MRONJ among students and practitioners. Therefore, these findings suggest increased emphasis should be placed on educating dentists and students about this condition to ensure patients receive the best possible care.

## Introduction

Bisphosphonate (BP) is a drug similar to pyrophosphate that has been prescribed since 1960 to treat various bone diseases [1-4]. It has been shown that BP causes the inhibition of osteoclasts, resulting in the reduction of bone resorption and bone remodelling, which leads to osteonecrosis of the jaw (ONJ), later known as BP-related osteonecrosis of the jaw (BRONJ) [5,6]. It was first reported in 2003 by Marx [7], and subsequent cases have been extensively reported in the scientific literature, leaving a significant impact on quality of life and substantial morbidity [8-12]. It appears as a bone exposed for eight weeks or more without any history of radiation therapy [4,13]. In 2014, the American Association of Oral and Maxillofacial Surgeons (AAOMS) modified the old terminology to medication-related osteonecrosis of the jaw (MRONJ) because of cases of ONJ related with other antiresorptive medications [5].

The etiology of MRONJ is not entirely understood, but there are several suggested risk factors which include the length of time a patient has been on BP therapy, method of administration, age of the patient, history of dentoalveolar surgery, use of corticosteroids, and presence of systemic disease like diabetes mellitus [4,14]. There has been a gradual increase in the occurrence of complications associated with the use of these drugs. The understanding of the mechanisms behind MRONJ is still lacking, and various hypotheses have been proposed to explain why MRONJ explicitly affects the jaws. The hypotheses proposed involve various factors that may contribute to the observed effects. These factors include the excessive suppression of bone resorption, changes in bone remodelling processes, ongoing microtrauma, inhibition of angiogenesis, vitamin D deficiency, suppression of acquired or innate immunity, presence of infection or inflammation, and potential toxicity of soft tissue blood pressure [4].

In MRONJ, bone resorption and remodelling decrease as osteoclast differentiation and function are inhibited and apoptosis increases. In all skeletal bones, osteoclasts play a crucial role in bone remodelling and healing. Conversely, ONJ occurs in the mandible 73% of the time and in the maxilla 22.5% of the time [15,16]. This phenomenon may be explained by the higher remodelling rate of the jaws compared to other skeletal bones. ONJ has been proven to be triggered by infection and inflammation in numerous clinical studies. Biopsy samples of necrotic bone taken from patients with ONJ have been found to contain bacteria, specifically *Actinomyces* spp. [4].

MRONJ is mostly a drug-related disease, with risk factors including dosage, administration method, duration, and therapeutic use. Other risk factors for this disease include surgical operations, such as tooth extraction; individuals with comorbidities, such as diabetes; and concurrent use of corticosteroids. Some low-risk cancer patients are given treatment for noncancer conditions such as osteoporosis, osteopenia, and Paget's disease [17].

It has been found that cancer-free individuals without having any additional risk who receive oral antiresorptive for less than four years have a relatively low risk of developing this disease. Low-risk patients can receive dental treatment without any modifications. However, cancer patients face a significant risk in developing multiple myeloma and bone metastases [18,19].

The treatment of MRONJ involves a comprehensive approach that includes prevention, ongoing cancer care, preservation of bone health, and enhancing the quality of life for patients. Strategies involve taking proactive measures to prevent MRONJ, ensuring that individuals on specific therapies can continue their oncologic treatments without interruption, and prioritizing bone health to minimize the risk of fractures. In addition, patient education plays a crucial role in empowering individuals to actively participate in their care. Pain management, infection control, and preventive measures to stop the progression of lesions in the jaw are essential for improving comfort and minimizing potential complications [4].

Dentists have a significant impact on preventing BRONJ and MRONJ by offering preventive care and prioritizing preventive treatment before starting BP [4,11,20,21]. Therefore, dentists and physicians must possess sufficient knowledge about identifying potential complications and the appropriate treatment for patients who are at risk of MRONJ [21].

Guidelines for patients getting BPs on staging and treatment approaches were released by the AAOMS. These guidelines' primary goal was to give physicians a foundational understanding of BPs, MRONJ/BRONJ clinical characteristics and risk factors, and, most importantly, how to treat and prevent MRONJ/BRONJ. Regretfully, investigations have revealed that dentists have shown very poor knowledge about the care of patients receiving BP therapy, even in spite of these guidelines [5,6].

There have been limited studies exploring dental students' knowledge and awareness about MRONJ. However, no study has been conducted thus far to investigate their understanding of drugs associated with it. Therefore, the objective of this study was to analyse and assess knowledge about MRONJ among dental students and practitioners in the central region of Saudi Arabia.

## Materials and methods

An observational cross-sectional study was planned to collect data from dental students and dentists in the central region of Saudi Arabia. To collect information from participants, a valid and reliable questionnaire was used [22] using a convenient non-probability sampling method during the period from October to December 2022. The Epi Info software (Centers for Disease Control and Prevention, Atlanta, Georgia, United States) was used to calculate the sample size, assuming a 50% incidence rate with a margin of error of 5% and a 95% level of confidence. The minimum sample required was 384; because of time constraints, we were able to collect responses from 250 participants. This study was approved by the Committee of Research Ethics of Qassim University (approval number: 21-12-03).

Inclusion/exclusion criteria 

Dental students, graduates, and dental practitioners were included, whereas the general public, medical practitioners, and medical students were excluded from participation. 

Data collection method 

This study utilized a survey divided into five components (see Appendices section). There were six items in the first section of the questionnaire pertaining to demographic information, namely, age, gender, college affiliation (graduated from or currently enrolled in), years of professional experience, and highest educational degree attained. The second component consisted of five items designed to assess participants' general knowledge of antiresorptive medications. The third component assessed participants' understanding of the therapeutic applications of antiresorptive and antiangiogenic drugs. In the fourth component, participants were assessed on their knowledge of the correct definition and the associated risk factors. The fifth section addressed the dental management of patients taking BPs.

Statistical analysis 

The data were analysed using IBM SPSS Statistics for Windows, Version 22.0 (Released 2013; IBM Corp., Armonk, New York, United States). Age, gender, marital status, and educational background were represented as frequencies and percentages. A chi-squared test was applied to determine the association between dentists' and students' knowledge related to ONJ. Statistical significance was determined by a p-value of less than 0.05.

## Results

A total of 250 participants were enrolled in the study. Of them, 128 (51.2%) were women, and 122 (48.8%) were men. Marital status revealed that most participants were single (198 or 79.2%) and 47 (18.8%) were married. Most participants (149 or 59.6%) were between the ages of 18 and 25, 82 (32.8%) were between the ages of 26 and 35, 13 (5.2%) were between the ages of 36 and 45, and only six (2.4%) were between the ages of 46 and 55. The colleges of dentistry at Qassim University, King Saud University, and Riyadh Elm University had 59 (23.6%), 55 (22%), and 39 (15.6%) participants, respectively. Additionally, 114 (45.6%) were students, whereas 136 (54.4%) were dentists, including dental interns, general practitioners, and specialists. Only 28 (11.2%) held a postgraduate degree (master's or PhD), as shown in Table 1. 

**Table 1:** Frequency distribution of demographic details of participants (n=250)

Variable		% (n)
Age (years)	18-25	59.6% (149)
	26-35	32.8% (82)
	36-45	5.2% (13)
	46-55	2.4% (6)
Gender	Male	48.8% (122)
	Female	51.2% (128)
Marital status	Married	18.8% (47)
	Single	79.2% (198)
	Separated	2% (5)
University	Qassim University	23.6% (59)
	King Saud University	22% (55)
	King Saud bin Abdulaziz University for Health Sciences	6% (15)
	Prince Sultan bin Abdulaziz University	8.4% (21)
	Riyadh Elm University	15.6% (39)
	Others	24.4% (61)
Educational level	Student	45.6% (114)
	Graduate	54.4% (136)
Enrolled year in college	First year	7.2% (18)
	Second year	7.6% (19)
	Third year	6% (15)
	Fourth year	5.2% (13)
	Fifth year	14.8% (37)
	Intern	4.8% (12)
	Graduated	54.4% (136)
Highest degree obtained	Student	45.6% (114)
	Bachelor	39.2% (98)
	Masters	5.6% (14)
	PhD	5.6% (14)
	Others	4% (10)

The general knowledge of antiresorptive/antiangiogenic medications revealed that most of the dentists (119 or 87.5%) knew about BP drugs as compared to students (78 or 68.4%), with a significant difference found among them (p<0.05). Almost all of the dentists (121 or 89%) and about 81 (71.1%) students thought it was important to ask patients about their usage of antiresorptive/antiangiogenic medications, with a significant difference found between them (p=0.05). It was observed that the university was the primary source of information for both the dentists (97 or 71.3%) and students (70 or 61.4%). Regarding obtaining knowledge via variable additional sources (such as scientific journals and medical meetings), the dentists' group had a higher tendency to obtain knowledge than the students' group (p=0.136). Most of the dentists (117 or 86%) and 69 (60.5%) students believed BPs can lead to ONJ, with a significant difference between them (p<0.05). Furthermore, most dentists (115 or 84.6%) and only 79 (23.7%) students thought that patients should be checked by a dentist before starting intravenous BP treatment, with a significant difference among them (p=0.05), as shown in Table 2. 

**Table 2:** General knowledge of bisphosphonates

Variables	Student (n=114) % (n)	Dentists (n=136) % (n)	p-value
Do you know bisphosphonate drugs?			
Yes	68.4% (78)	87.5% (119)	<0.05
No	31.6% (36)	12.5% (17)	
Is it important to ask patients about use of bisphosphonate medications?			
Yes	71.1% (81)	89% (121)	0.05
No	2.6% (3)	1.5% (2)	
I don't know	26.3% (30)	9.6% (13)	
Where have you heard about bisphosphonate medications?			
University	61.4% (70)	71.3% (97)	0.136
Mass media	7% (8)	1.5% (1)	
Scientific journals	9.6% (11)	10.3% (14)	
Medical meetings	5.3% (6)	5.1% (7)	
Never heard	16.7% (19)	11.8% (16)	
Can bisphosphonates lead to osteonecrosis of the jaw?			
Yes	60.5% (69)	86% (117)	<0.05
No	11.4% (13)	5.1% (7)	
I don't know	28.1% (32)	8.8% (12)	
Examination of patient before starting an IV bisphosphonate treatment			
Yes	23.7% (79)	84.6% (115)	0.05
No	7% (8)	1.5% (2)	
I don't know	69.3% (27)	14% (19)	

The study found that there was a general lack of knowledge regarding the therapeutic uses of antiresorptive and antiangiogenic medications in both dentists and students. Importantly, there were no significant differences between the two groups in terms of their knowledge (p=0.552). The data reveal that bone metastasis is the most commonly recognized therapeutic use of antiresorptive therapy among students, accounting for 25 (21.9%) of the responses. Furthermore, dentists primarily associate antiresorptive therapy with treating osteopenia and osteoporosis, which accounted for 28 (20.6%) of the responses. Interestingly, 62 (54.4%) students and 42 (30.9%) dentists could not identify BPs' active principle or commercial name. Out of all the listed BP medications, alendronate (Fosamax, Merck & Co., Rahway, New Jersey, United States) was the most recognized, followed by zoledronate (Zometa, Novartis, Basel, Switzerland), with a significant difference between them (p<0.05). Most of the dentists (77 or 56.6%) and students (73 or 64%) did not know that any other medications could lead to ONJ, with an insignificant difference among them (p=0.288), as shown in Table 3. 

**Table 3:** Knowledge and therapeutic uses of medications

Variables	Student (n=114) % (n)	Dentists (n=136) % (n)	p-value
What are the pathology target of a bisphosphonate therapy?			
Bone metastases	21.9% (25)	14% (19)	0.552
Chondroblastoma	2.6% (3)	1.5% (2)	
Hypercalcemia of malignancy	2.6% (3)	2.2% (3)	
Multiple myeloma	7% (8)	8.1% (11)	
Osteogenesis imperfecta	5.3% (6)	4.4% (6)	
Osteomyelitis	13.2% (15)	14% (19)	
Osteopenia and osteoporosis	14.9% (17)	20.6% (28)	
Osteopetrosis	12.3% (14)	8.1% (11)	
Paget's disease of bone	8.8% (10)	11.8% (16)	
All of the above	11.4% (13)	15.4% (21)	
Do you know the active principle and commercial name of bisphosphonates?			
Alendronate (Fosamax, Merck & Co., Rahway, New Jersey, United States)	17.5% (20)	22.8% (31)	<0.05
Ibandronate (Boniva, Roche, Basel, Switzerland)	10.5% (12)	5.1% (7)	
Neridronate (Nerixia, Abiogen Pharma, Pisa, Italy)	4.4% (5)	2.9% (4)	
Pamidronate (Aredia, Pfizer, New York, New York, United States)	3.5% (4)	2.2% (3)	
Risedronate (Actonel and Atelvia, Greenstone Pharma, North America, United States)	7% (8)	11.8% (16)	
Tiludronate (Skelid, Sanofi, Paris, France)	0% (0)	1.5% (2)	
Zoledronate (Zometa, Novartis, Basel, Switzerland)	2.6% (3)	22.8% (31)	
None	54.4% (62)	30.9% (42)	
Do you know any other medications involved in the osteonecrosis of the jaw?			
Bevacizumab (Avastin, Roche, Basel, Switzerland)	7% (8)	10.3% (14)	0.288
Denosumab (Xgeva and Prolia, Amgen, Thousand Oaks, California, United States)	13.2% (15)	20.6% (28)	
Sirolimus (Rapamune, Pfizer, New York, New York, United States)	2.6% (3)	4.4% (6)	
Sorafenib (Nexavar, Bayer, Berlin, Germany)	6.1% (7)	5.1% (7)	
Sunitinib (Sutent, Pfizer, New York, New York, United States)	7% (8)	2.9% (4)	
I don't know any non-bisphosphonate drug	64% (73)	56.6% (77)	

Regarding knowledge of the correct definition of ONJ, only a small proportion of dentists (30 or 22.1%) and students (25 or 21.9%) knew the correct definition of MRONJ according to the AAOMS, but an insignificant difference was observed among them (p=0.779). Regarding the risk factors of MRONJ, tobacco was the most recognized by 28 (20.6%) dentists and 19 (16.7%) students, with an insignificant association among them (p=0.409) as shown in Table 4. 

**Table 4:** Knowledge about risk factors and correct definition of MRONJ MRONJ: medication-related osteonecrosis of the jaw

Variables	Student (n=114) % (n)	Dentists (n=136) % (n)	p-value
Risk factors of osteonecrosis of the jaw			
Alcohol	8.8% (10)	11% (15)	0.409
Antibiotic therapy	10.5% (12)	2.9% (4)	
Arterial hypertension	1.8% (2)	0.7% (1)	
Hyperlipidemia	2.6% (3)	2.2% (3)	
Length of therapy	1.8% (2)	5.9% (8)	
Microtrauma	7.9% (9)	7.4% (10)	
Steroid therapy	1.8% (2)	2.9% (4)	
Tobacco	16.7% (19)	20.6% (28)	
Tobacco, alcohol, length of therapy, steroid therapy	17.5% (20)	14% (19)	
Total amount of drug administered	1.8% (2)	0.7% (1)	
Way of administration	7% (8)	5.1% (7)	
Way of administration, length of therapy, steroid therapy	3.5% (4)	5.1% (7)	
All of the above	18.4% (21)	21.3% (29)	
Percentage of correct MRONJ definition			
Bone exposed to the maxillofacial region that is visible through an intraoral or extraoral fistula or has persisted for at least eight weeks in patients who are currently or have been treated with antiresorptive or antiangiogenic agents and who have no history of radiation therapy or metastatic disease in the jaws	21.9% (25)	22.1% (30)	0.779

Regarding the level of knowledge about the dental management of patients receiving BP therapy, most dentists (80 or 58.8%) and 58 students (50.9%) did not think invasive dental treatment could be performed safely on patients during intravenous BP therapy. In comparison, 32 (23.5%) dentists and 10 (8.8%) students thought that patients on intravenous BP therapy could possibly undergo invasive dental procedures without risk, with a significant difference between them (p<0.05). Conversely, there was an insignificant difference observed between dentists and students that patients who are on oral BP therapy for a duration of less than four years and in the absence of any risk factors could safely undergo invasive dental treatment (p=0.186). Additionally, 47 (34.6%) dentists and 39 (34.2%) students recognized that taking oral BP therapy less than four years will make invasive dental treatment unsafe for such patients, with an insignificant difference observed between dentists and students (p=0.851). In addition, 42 (30.9%) dentists and 20 (17.5%) students indicated that for patients who are on oral BP therapy for more than four years, invasive dental treatment could be performed safely, with a significant difference observed between dentists and students (p=0.048). Most of the dentists (111 or 81.6%) and 91 (79.8%) students wanted to learn more about the ONJ; however, an insignificant association was noticed between them (p=0.913), as shown in Table 5.

**Table 5:** Knowledge about the dental management of patients on bisphosphonate therapy

Variables	Student (n=114) % (n)	Dentists (n=136) % (n)	p-value
Can intravenous bisphosphonate patients receive invasive dental procedures?			
Yes	8.8% (10)	23.5% (32)	<0.05
No	50.9% (58)	58.8% (80)	
I don't know	40.4% (46)	17.6% (24)	
Are invasive dental procedures safe for patients taking oral bisphosphonates for less than four years without risk factors?			
Yes	20.2% (23)	29.4% (40)	0.186
No	31.6% (36)	31.6% (43)	
I don't know	48.2% (55)	39% (53)	
Are invasive dental treatments safe for people with risk factors who have been taking oral bisphosphonates for <4 years?			
Yes	20.2% (23)	22.8% (31)	0.851
No	34.2% (39)	34.6% (47)	
I don't know	45.6% (52)	42.6% (58)	
Can oral bisphosphonate users over four years safely undergo invasive dental procedures?			
Yes	17.5% (20)	30.9% (42)	0.048
No	32.5% (37)	27.9% (38)	
I don't know	60% (57)	41.2% (56)	
Do you want to learn more about jaw osteonecrosis?			
Yes	79.8% (91)	81.6% (111)	0.931
No	6.1% (7)	5.9% (8)	
I don't know	14% (16)	12.5% (17)	

## Discussion

MRONJ is a significant and debilitating adverse medication reaction observed in individuals undergoing prolonged treatment with antiresorptive or antiangiogenic drugs, primarily impacting the mandible more commonly than the maxilla. Having a sufficient understanding of MRONJ is essential to enhance treatment results and mitigate the problems linked to these drugs. Based on the available information, a scant amount of research has investigated the extent of knowledge of MRONJ among dental healthcare providers and dentistry students.

This study was conducted to evaluate the knowledge of dentists and students regarding MRONJ to improve patient care. In the sample of 250 participants, this study found that most of the dentists knew about BP drugs (87.5%) and this is in alignment with previous research conducted in Saudi Arabia. However, both studies by Almousa et al. and Al-Eid et al. revealed a comparatively lower level of knowledge among dentists, with percentages of 66.5% and 60.8%, respectively. Furthermore, comparable findings were observed in previous studies conducted among dental professionals, indicating that 70% of dentists were aware of MRONJ. In a separate investigation, it was discovered that 83.3% of dental professionals and 99% of students reported possessing knowledge of BPs [22,23]. In addition, similar results were found in Al-Maweri et al.'s study conducted among dentists, which showed that 70% knew about MRONJ [24]. Other studies found 83.3% of dentists and 99% of students declared to know BPs [18,25].

University teaching was the primary source of knowledge attained by dentists (71.3%) and students (61.4%). In general, the group of dentists tended to gain knowledge from many external sources, including the media, scientific journals, and professional gatherings, in contrast to the student group. One possible explanation for this phenomenon is that the dentist group may have a higher likelihood of seeing patients at risk of MRONJ and actively engaging in continuing medical education (CME) programs. The data indicate 89% of dentists consider it necessary to inquire about patients' use of antiresorptive/antiangiogenic medications, in contrast to 71.1% of students. The overall knowledge of dentists and students with regard to the therapeutic uses of antiresorptive and antiangiogenic medications was low. The most common therapeutic use recognized by students was bone metastasis (21.9%), whereas osteopenia and osteoporosis (20.6%) were also recognized by dentists.

In the current study, it was observed that a significant portion of the participants lacked knowledge about the specific antiresorptive medications despite the inclusion of both the generic and brand names of these medications. The study conducted by de Lima et al. and Almousa et al. among dentists and dental students revealed similar results, indicating that most (86%) participants could not identify the commercial brand names of BP medication [20,22].

Regarding BP side effects, a study revealed that alendronate (Fosamax) and zoledronate (Zometa) can cause osteonecrosis, whereas a large number of dentists (56.6%) and students (64%) were unaware that other medications might induce ONJ. The difference in knowledge between these two groups was statistically insignificant. Rosella et al. and Almousa et al. have reported similar findings regarding the impact of well-known BP medications [22,26].

The process of identifying medications is crucial to minimizing the potential of providing care without fully understanding the associated risks.

Regarding knowledge of the precise definition of ONJ, only a small proportion of dentists (22.1%) and students (21.9%) possessed this knowledge. The AAOMS defines MRONJ as the presence of exposed bone or bone that can be probed through a fistula in the maxillofacial region that persists for more than eight weeks in patients who have been treated with antiresorptive or antiangiogenic agents, without a history of radiation therapy to the jaws or evident metastatic disease in the jaws [4]. The results demonstrate similarity with Almousa et al.'s, Al-Eid et al.'s, and Al-Maweri et al.'s studies, emphasizing the limited understanding of the clinical characteristics of MRONJ [22-24]. On the contrary, Spanish dentists and dental students exhibited more significant levels of knowledge because of their greater familiarity with the accurate definition of this disease, as reported by López-Jornet et al. [27]. Lack of understanding about the definition can lead to delayed detection or unwarranted treatments, thereby heightening the likelihood of more serious complications.

The participants' responses regarding the risk factors were inadequate because less than 50% of them correctly identified the risk factors. According to the data, a significant percentage of dentists (58.8%) and students (50.9%) hold the belief that invasive dental procedures may not be safe for patients undergoing intravenous BP therapy. The data indicate that a higher percentage of dentists (23.5%) compared to students (8.8%) believed that the task could be carried out without any risks. The findings from our study are consistent with the results of Almousa et al.'s study [22].

Overall, the findings indicate a lack of awareness about patient management, resulting in deferring necessary treatment when the risk is low while attempting high-risk treatments without taking the appropriate precautions.

The present study acknowledges several limitations. The sample size was relatively small and limited to specific locations in Saudi Arabia. The outcomes reported may lack representativeness for dentists nationwide. Future studies should include larger sample sizes and broaden their sampling to include other regions in Saudi Arabia.

## Conclusions

The implications of the findings in the present study warrant increased emphasis on the importance of educating students and dentists about this disease. It is highly recommended to attend continuing education courses that focus on treating and preventing this ailment in patients undergoing BP therapy.

## Figures and Tables

**Figure 1:**
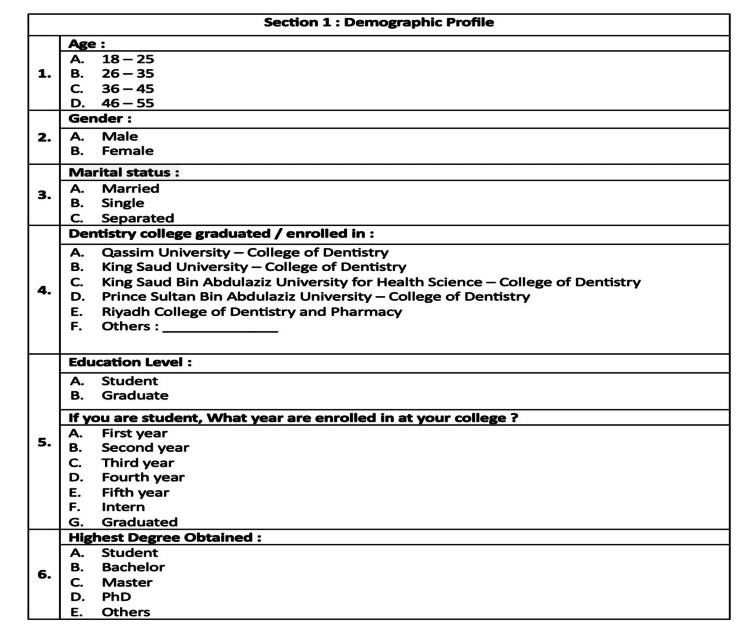
Questionnaire (Section 1)

**Figure 2:**
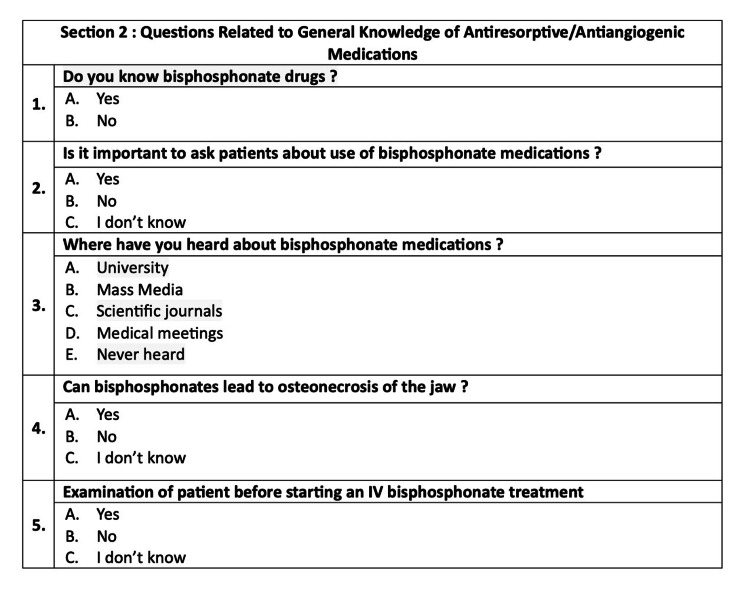
Questionnaire (Section 2)

**Figure 3:**
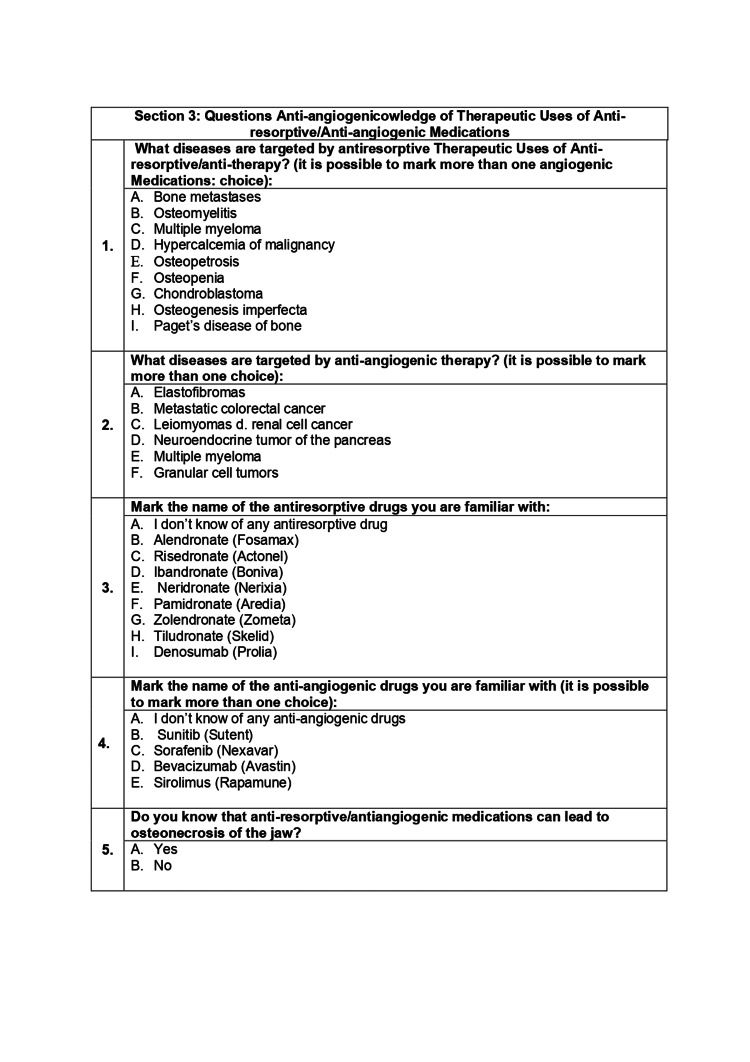
Questionnaire (Section 3)

**Figure 4:**
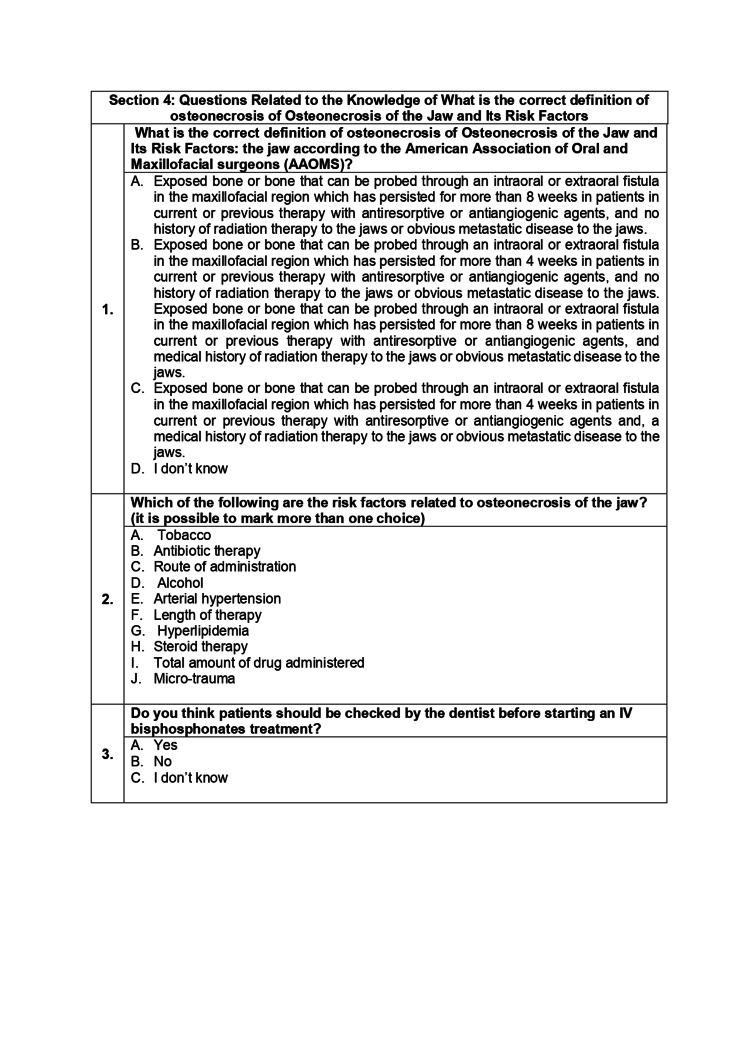
Questionnaire (Section 4)

**Figure 5:**
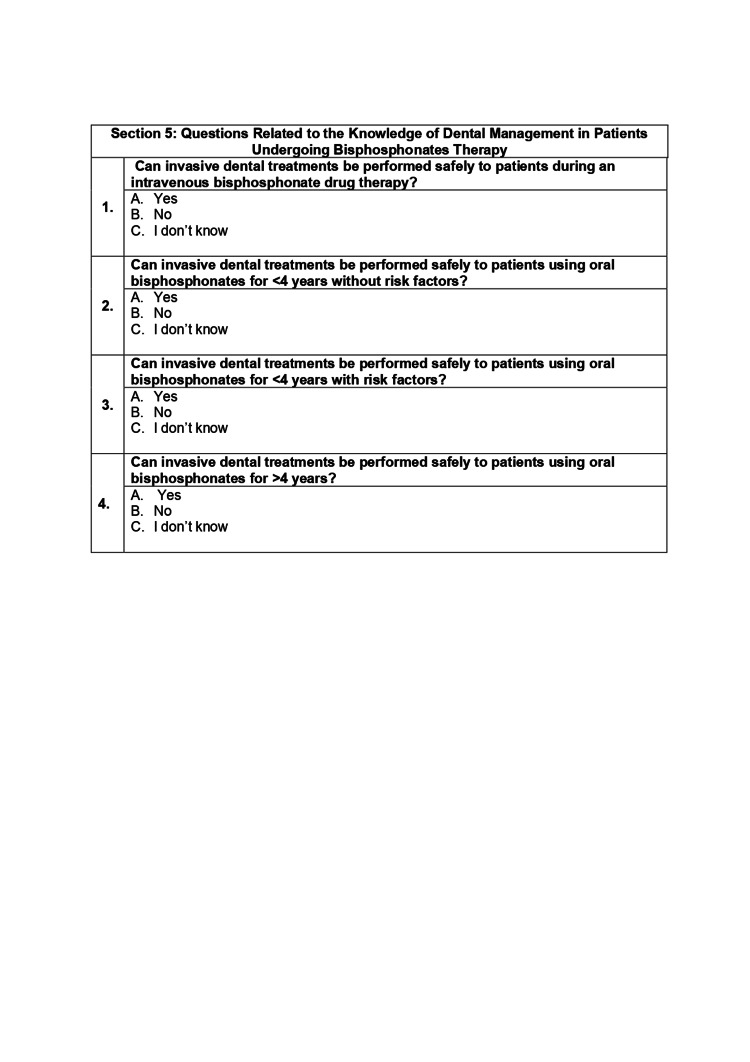
Questionnaire (Section 5)

## References

[1] Viviano M, Addamo A, Cocca S (2017). A case of bisphosphonate-related osteonecrosis of the jaw with a particularly unfavourable course: a case report. J Korean Assoc Oral Maxillofac Surg.

[2] Reyes C, Hitz M, Prieto-Alhambra D, Abrahamsen B (2016). Risks and benefits of bisphosphonate therapies. J Cell Biochem.

[3] Vinitzky-Brener I, Ibáñez-Mancera NG, Aguilar-Rojas AM, Álvarez-Jardón AP (2017). Knowledge of bisphosphonate-related osteonecrosis of the jaws among Mexican dentists. Med Oral Patol Oral Cir Bucal.

[4] Ruggiero SL, Dodson TB, Aghaloo T, Carlson ER, Ward BB, Kademani D (2022). American Association of Oral and Maxillofacial Surgeons' position paper on medication-related osteonecrosis of the jaws-2022 update. J Oral Maxillofac Surg.

[5] Ruggiero SL, Dodson TB, Fantasia J, Goodday R, Aghaloo T, Mehrotra B, O'Ryan F (2014). American Association of Oral and Maxillofacial Surgeons position paper on medication-related osteonecrosis of the jaw--2014 update. J Oral Maxillofac Surg.

[6] Ruggiero SL, Dodson TB, Assael LA, Landesberg R, Marx RE, Mehrotra B (2009). American Association of Oral and Maxillofacial Surgeons position paper on bisphosphonate-related osteonecrosis of the jaws--2009 update. J Oral Maxillofac Surg.

[7] Marx RE (2003). Pamidronate (Aredia) and zoledronate (Zometa) induced avascular necrosis of the jaws: a growing epidemic. J Oral Maxillofac Surg.

[8] Levin L, Laviv A, Schwartz-Arad D (2007). Denture-related osteonecrosis of the maxilla associated with oral bisphosphonate treatment. J Am Dent Assoc.

[9] Lee JJ, Cheng SJ, Jeng JH, Chiang CP, Lau HP, Kok SH (2011). Successful treatment of advanced bisphosphonate-related osteonecrosis of the mandible with adjunctive teriparatide therapy. Head Neck.

[10] Bedogni A, Bettini G, Totola A, Saia G, Nocini PF (2010). Oral bisphosphonate-associated osteonecrosis of the jaw after implant surgery: a case report and literature review. J Oral Maxillofac Surg.

[11] Gil IG, Ponte BM, Mateo ST, García JJ (2019). Treatment of bisphosphonate-related osteonecrosis of the jaw with plasma rich in growth factors after dental implant surgery: a case report. J Oral Implantol.

[12] Suzuki N, Oguchi H, Yamauchi Y, Karube Y, Suzuki Y, Hosoya N (2017). A case of tooth fracture occurred upon medicating bisphosphonate for an elderly person: preservation therapy and responses for stage 0 of bisphosphonate-related osteonecrosis of jaw. Eur J Dent.

[13] Thumbigere-Math V, Sabino MC, Gopalakrishnan R (2009). Bisphosphonate-related osteonecrosis of the jaw: clinical features, risk factors, management, and treatment outcomes of 26 patients. J Oral Maxillofac Surg.

[14] Awad ME, Sun C, Jernigan J, Elsalanty M (2019). Serum C-terminal cross-linking telopeptide level as a predictive biomarker of osteonecrosis after dentoalveolar surgery in patients receiving bisphosphonate therapy: systematic review and meta-analysis. J Am Dent Assoc.

[15] Pazianas M, Miller P, Blumentals WA, Bernal M, Kothawala P (2007). A review of the literature on osteonecrosis of the jaw in patients with osteoporosis treated with oral bisphosphonates: prevalence, risk factors, and clinical characteristics. Clin Ther.

[16] Kang MH, Lee DK, Kim CW, Song IS, Jun SH (2018). Clinical characteristics and recurrence-related factors of medication-related osteonecrosis of the jaw. J Korean Assoc Oral Maxillofac Surg.

[17] Beth-Tasdogan NH, Mayer B, Hussein H, Zolk O (2017). Interventions for managing medication-related osteonecrosis of the jaw. Cochrane Database Syst Rev.

[18] Rosella D, Papi P, Giardino R, Cicalini E, Piccoli L, Pompa G (2016). Medication-related osteonecrosis of the jaw: clinical and practical guidelines. J Int Soc Prev Community Dent.

[19] Conte P, Guarneri V (2004). Safety of intravenous and oral bisphosphonates and compliance with dosing regimens. Oncologist.

[20] Alhussain A, Peel S, Dempster L, Clokie C, Azarpazhooh A (2015). Knowledge, practices, and opinions of ontario dentists when treating patients receiving bisphosphonates. J Oral Maxillofac Surg.

[21] de Lima PB, Brasil VL, de Castro JF, de Moraes Ramos-Perez FM, Alves FA, dos Anjos Pontual ML, da Cruz Perez DE (2015). Knowledge and attitudes of Brazilian dental students and dentists regarding bisphosphonate-related osteonecrosis of the jaw. Support Care Cancer.

[22] Almousa MA, Alharbi GK, Alqahtani AS, Chachar Y, Alkadi L, Aboalela A (2021). Dental practitioners' and students' knowledge of medication related osteonecrosis of the jaw (MRONJ). Saudi Pharm J.

[23] Al-Eid R, Alduwayan T, Bin Khuthaylah M, Al Shemali M (2020). Dentists' knowledge about medication-related osteonecrosis of the jaw and its management. Heliyon.

[24] Al-Maweri SA, Alshammari MN, Alharbi AR (2020). Knowledge and opinions of Saudi dentists regarding dental treatment of patients undergoing bisphosphonates. Eur J Dent.

[25] Patil V, Acharya S, Vineetha R, Nikhil K (2020). Awareness about medication-related osteonecrosis of the jaw among dental professionals: a multicentre study. Oral Health Prev Dent.

[26] Rosella D, Papi P, Pompa G, Capogreco M, De Angelis F, Di Carlo S (2017). Dental students' knowledge of medication-related osteonecrosis of the jaw. Eur J Dent.

[27] López-Jornet P, Camacho-Alonso F, Molina-Miñano F, Gomez-Garcia F (2010). Bisphosphonate-associated osteonecrosis of the jaw. Knowledge and attitudes of dentists and dental students: a preliminary study. J Eval Clin Pract.

